# Analysis of the anti-apoptotic activity of four vaccinia virus proteins demonstrates that B13 is the most potent inhibitor in isolation and during viral infection

**DOI:** 10.1099/vir.0.068833-0

**Published:** 2014-12

**Authors:** David L. Veyer, Carlos Maluquer de Motes, Rebecca P. Sumner, Louisa Ludwig, Benjamin F. Johnson, Geoffrey L. Smith

**Affiliations:** 1 Department of Pathology, University of Cambridge, Cambridge CB2 1QP, UK; 2 Equipe Microbiologie, EA 1254, SFR BIOSIT, Université Européenne de Bretagne, Rennes, France; 3 Virology Laboratory, Pontchaillou University Hospital, Rennes 35033, France; 4 Department of Virology, Imperial College London, London W2 1PG, UK

## Abstract

Vaccinia virus (VACV) is a large dsDNA virus encoding ~200 proteins, several of which inhibit apoptosis. Here, a comparative study of anti-apoptotic proteins N1, F1, B13 and Golgi anti-apoptotic protein (GAAP) in isolation and during viral infection is presented. VACVs strains engineered to lack each gene separately still blocked apoptosis to some degree because of functional redundancy provided by the other anti-apoptotic proteins. To overcome this redundancy, we inserted each gene separately into a VACV strain (vv811) that lacked all these anti-apoptotic proteins and that induced apoptosis efficiently during infection. Each protein was also expressed in cells using lentivirus vectors. In isolation, each VACV protein showed anti-apoptotic activity in response to specific stimuli, as measured by immunoblotting for cleaved poly(ADP ribose) polymerase-1 and caspase-3 activation. Of the proteins tested, B13 was the most potent inhibitor, blocking both intrinsic and extrinsic stimuli, whilst the activity of the other proteins was largely restricted to inhibition of intrinsic stimuli. In addition, B13 and F1 were effective blockers of apoptosis induced by vv811 infection. Finally, whilst differences in induction of apoptosis were barely detectable during infection with VACV strain Western Reserve compared with derivative viruses lacking individual anti-apoptotic genes, several of these proteins reduced activation of caspase-3 during infection by vv811 strains expressing these proteins. These results illustrated that vv811 was a useful tool to determine the role of VACV proteins during infection and that whilst all of these proteins have some anti-apoptotic activity, B13 was the most potent.

## Introduction

Cell death is an essential biological process for development, cellular homeostasis and immune regulation, and can also restrict virus replication. Cell death occurs most commonly by apoptosis – an irreversible cascade of proteolytic events induced by the extrinsic or intrinsic activation of caspase proteases, particularly caspase-3 (Tait & Green, 2010). Extrinsic, or death receptor-mediated apoptosis, is initiated by Fas ligand or TNF, and induces the dimerization and activation of pro-caspase-8, which activates the effector caspases. Intrinsic, or mitochondrial apoptosis, is triggered by stimuli such as cell-cycle dysregulation, DNA damage or pathogen sensing, and causes mitochondrial outer membrane permeabilization (MOMP) and cytochrome *c* release, which, together with the apoptotic protease activating factor (APAF)-1 and caspase-9, form the apoptosome complex (Tait & Green, 2010). A tight regulation preceding MOMP occurs through a complex protein–protein interaction network involving the B-cell lymphoma (Bcl) family of proteins, which contains anti-apoptotic members, such as Bcl-2 and Bcl-x_L_, and pro-apoptotic members, such as Bax and Bak, and the BH3-only proteins Bid, Bad and Bim. The homo-oligomerization of the former is a pivotal step that triggers MOMP.

Apoptosis leads to morphological changes to cells and production of immunosuppressive cytokines (Griffith & Ferguson, 2011). In contrast, cell death deriving from caspase-1 activation and IL-1β production, termed pyroptosis, triggers inflammation. A third form of cell death called necroptosis occurs without caspase activity, but via the association of receptor interacting protein (RIP)-1 and -3 (Holler *et al.*, 2000), and is the default route of cell death when the apoptotic signal cannot proceed canonically due to inhibition of caspase activity (Han *et al.*, 2011). All forms of programmed cell death may be induced upon pathogen infection as part of innate immunity and consequently pathogens have evolved strategies to prevent their activation.

*Vaccinia virus* (VACV) is a member of the genus *Orthopoxvirus*. The centre of the VACV genome encodes proteins required for replication, whilst the terminal regions encode scores of proteins aiding immune evasion (Gubser *et al.*, 2004; Smith *et al.*, 2013), including apoptosis (for reviews, see Shisler & Moss, 2001; Taylor & Barry, 2006). Infection by VACV strain Western Reserve (WR) does not trigger apoptosis in many cultured cells and apoptotic pathways remained blocked (Lee & Esteban, 1994; Dobbelstein & Shenk, 1996; Kettle *et al.*, 1997; Wasilenko *et al.*, 2001). However, VACV strain Copenhagen (COP) infection induced apoptosis more readily (Heinkelein *et al.*, 1996). In addition, mice infected with VACV activated necroptotic signalling to control infection in response to viral inhibition of caspase-3 (Cho *et al.*, 2009). This indicated that VACV counteracts apoptosis efficiently during infection both *in vitro* and in animals.

At least six different VACV proteins prevent apoptosis. The first described was B13, the orthologue of cowpox virus cytokine response modifier A (CrmA), also called serine protease inhibitor (SPI)-2 (Kotwal & Moss, 1989; Smith *et al.*, 1989b). B13 and CrmA (Enari *et al.*, 1995; Los *et al.*, 1995; Miura *et al.*, 1995; Tewari & Dixit, 1995) act as a pan-caspase inhibitor, and inhibit caspase-8-mediated extrinsic apoptosis and caspase-1-induced inflammasome activation (Dobbelstein & Shenk, 1996; Heinkelein *et al.*, 1996; Kettle *et al.*, 1997). A related serpin called SPI-1 encoded by B22R gene in VACV strain WR [equivalent to the C12L gene in VACV strain COP (Goebel *et al.*, 1990; Smith *et al.*, 1991)] also has anti-apoptotic activity (Ali *et al.*, 1994; Brooks *et al.*, 1995) and is more conserved among different VACV strains (Kettle *et al.*, 1995). Another VACV protein with anti-apoptotic activity in some cell types, including HeLa cells, is the dsRNA-binding protein E3 (Lee & Esteban, 1994; Kibler *et al.*, 1997; García *et al.*, 2002). More recently, the structures of VACV proteins F1 (Kvansakul *et al.*, 2008) and N1 (Aoyagi *et al.*, 2007; Cooray *et al.*, 2007) were solved, and revealed a Bcl-2 fold containing a surface groove similar to cellular anti-apoptotic Bcl-2 proteins. Protein F1 binds Bak and blocks mitochondrial apoptosis (Wasilenko *et al.*, 2003, 2005; Postigo *et al.*, 2006; Kvansakul *et al.*, 2008), whereas N1 binds the upstream BH3-only proteins Bad and Bid, and also has some anti-apoptotic activity (Cooray *et al.*, 2007; Maluquer de Motes *et al.*, 2011). Both F1 and N1 also have other functions. F1 binds the nucleotide-binding domain, leucine-rich repeat and pyrin domain-containing protein (NLRP)-1, reducing inflammasome activation and pyroptosis (Gerlic *et al.*, 2013). N1 inhibits activation of NFκB (DiPerna *et al.*, 2004; Cooray *et al.*, 2007; Maluquer de Motes *et al.*, 2011) and IFN regulatory factor (IRF)-3 (DiPerna *et al.*, 2004; Dai *et al.*, 2014). Structure-based mutagenesis of N1 showed that the surface groove is important for Bid and Bad binding, and for inhibition of apoptosis, whereas the interface of the N1 homodimer (Bartlett *et al.*, 2002) is important for NFκB inhibition (Maluquer de Motes *et al.*, 2011). Others reported N1 did not block apoptosis (Banadyga *et al.*, 2009; Postigo & Way, 2012). Lastly, a few VACV strains, and camelpox virus (Gubser & Smith, 2002), encode a Golgi anti-apoptotic protein (GAAP) that protect cells from apoptosis and has a highly conserved human orthologue (hGAAP) with anti-apoptotic activity (Gubser *et al.*, 2007). Viral GAAP and hGAAP are members of the transmembrane Bax inhibitor-containing motif (TMBIM) family, and are hydrophobic proteins with six transmembrane domains (Carrara *et al.*, 2012) that oligomerize (Saraiva *et al.*, 2013b), alter the calcium storage in intracellular organelles (de Mattia *et al.*, 2009), and affect cell adhesion and migration (Saraiva *et al.*, 2013a).

Identification of additional VACV anti-apoptotic proteins by knocking out candidate genes from VACV strains such as WR is difficult because the effects are masked by the other anti-apoptotic proteins. An alternative approach is ectopic expression of proteins outside of viral infection and addition of pro-apoptotic stimuli to trigger cell death. However, whether these conditions mimic those under which viral proteins have evolved is unlikely. VACV strain vv811 lacks 55 genes from the parental strain COP, has a small plaque phenotype and replicates poorly in cell culture (Perkus *et al.*, 1991). Genes missing include B13R, F1L and N1L, as well as GAAP (which is not present in COP), and consequently vv811 is unable to protect cells from apoptosis induced by pro-apoptotic stimulus (Wasilenko *et al.*, 2003). Here, the potency of four different VACV anti-apoptotic proteins in isolation and in the context of viral infection was assessed using recombinant vv811 viruses expressing B13, F1, N1 and GAAP. Apoptosis was induced using extrinsic and intrinsic stimuli, and also by vv811 infection that alone caused caspase-3 activation. Results showed that although all proteins prevented apoptosis to some extent in different environments, B13 provided the greatest protection both in isolation and during viral infection.

## Results

### Generation of U2-OS cells constitutively expressing VACV anti-apoptotic proteins

**Fig. 1.**
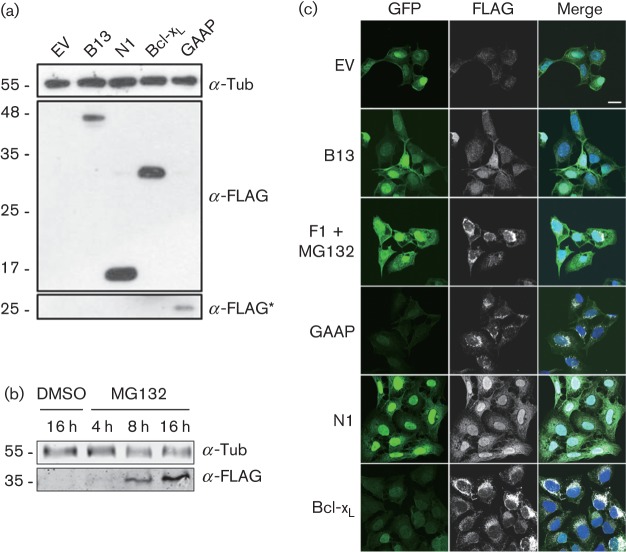
Expression of VACV anti-apoptotic proteins by lentivirus-transduced U2-OS cells. (a) Lysates of lentivirus-transduced U2-OS cells were analysed by SDS-PAGE and immunoblotting with indicated antibodies. A long exposure is indicated with an asterisk. (b) For F1 detection, the cells were treated with MG132 for the indicated time prior to immunoblotting. Tub, α-tubulin. Molecular size markers are indicated on the left (kDa). (c) Immunofluorescence. Cells were fixed and stained for immunofluorescence analysis using rabbit anti-FLAG antibody. The expression of GFP, FLAG-tagged proteins and merged images with DNA stained with DAPI are shown. Bar, 20 µm.

To study the anti-apoptotic capacity of individual VACV proteins, bi-cistronic lentivirus vectors were used to generate polyclonal U2-OS cell lines expressing GFP- and FLAG-tagged B13, F1, GAAP and N1 (Methods). An empty vector (EV) cell line expressing GFP only and a cell line expressing Bcl-x_L_ were also generated as controls. Expression of B13, GAAP, F1 and Bcl-x_L_ was verified by FLAG immunoblotting (Fig. 1a). Given its previously described degradation by the proteasome when expressed in isolation (Postigo *et al.*, 2006), F1 was only detected after pharmacological inhibition of the proteasome with MG132 (Fig. 1b). Analysis of these cells by immunofluorescence using an anti-FLAG antibody demonstrated the expression and cellular localization of these proteins (Fig. 1c). The location of each protein was as described previously, except that N1 showed a greater nuclear localization than noted elsewhere (Bartlett *et al.*, 2002; Maluquer de Motes *et al.*, 2011), possibly due to higher expression.

### Inhibition of extrinsic apoptosis in U2-OS cells expressing VACV anti-apoptotic proteins

**Fig. 2.**
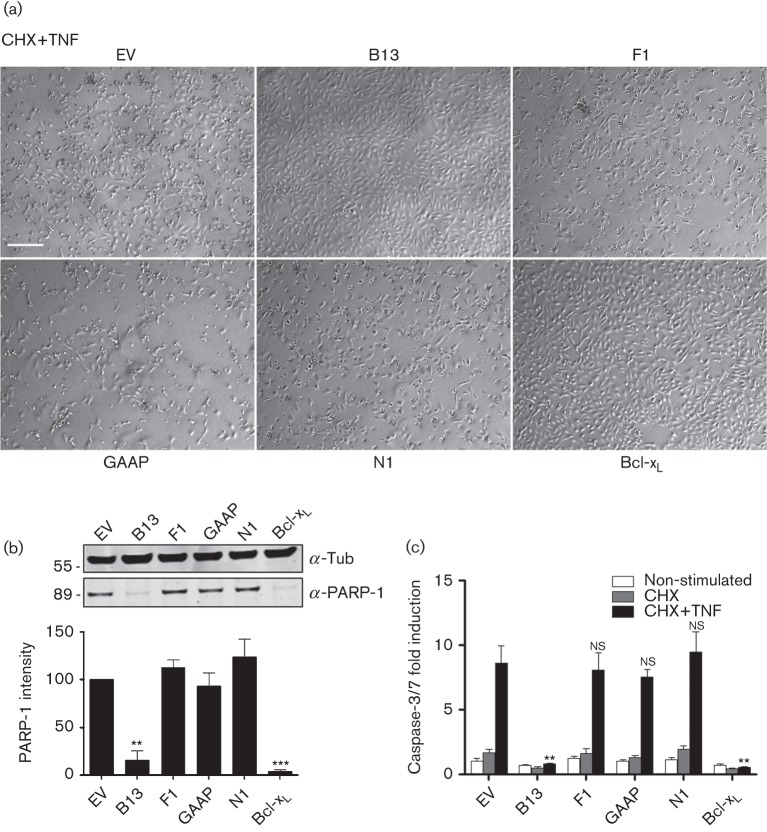
Induction of extrinsic apoptosis in the transduced U2-OS cells. (a) Cells were incubated overnight with CHX and TNF-α (10 µg ml^−1^) for 16 h and then photographed (see Fig. S1 for control). Bar, 200 µm. (b) Cells were incubated overnight with CHX and TNF-α (10 µg ml^−1^) for 16 h, and lysates were analysed by SDS-PAGE and immunoblotting against cleaved PARP-1 or α-tubulin (Tub). A representative blot of three repeats is shown. Quantitative data were obtained by integrating the intensity of the bands from the three repeats using a LI-COR system. Data were normalized to α-tubulin and are shown as the mean±sd. (c) Cells were incubated overnight with either CHX (30 µg ml^−1^) or CHX and TNF-α (10 µg ml^−1^) for 16 h, and apoptosis was assessed quantitatively using Caspase-Glo. Data are shown as the mean±sd fold induction relative to EV and are representative of at least three experiments each performed in triplicate. Statistical differences between EV and anti-apoptotic protein-transduced cells were determined using an unpaired Student’s *t*-test (*P<0.05, **P<0.01).

To assess anti-apoptotic activity, extrinsic apoptosis was induced using cycloheximide (CHX) and TNF-α. This induced cell death in EV-transduced cells, as seen by morphological changes and fewer adherent cells remaining, and this was prevented in cells expressing B13 or Bcl-x_L_ (Figs 2a and S1, available in the online Supplementary Material). Immunoblotting for cleaved poly(ADP ribose) polymerase (PARP)-1 confirmed induction of apoptosis in cells expressing F1, N1 and GAAP, and its inhibition by B13 or Bcl-x_L_ (Fig. 2b, upper), and this was quantified in three independent experiments (Methods) (Fig. 2b, lower). A second analysis was obtained using the quantitative Caspase-Glo 3/7 assay. TNF-α treatment caused ~8-fold increase in caspase-3 activity in EV-transduced cells, and this was reduced significantly only in cells expressing B13 and Bcl-x_L_ (Fig. 2c). These results indicated that out of these four VACV proteins, only B13 blocked TNF-α-induced apoptosis in U2-OS cells, consistent with its inhibition of caspase-8 cleavage.

### Inhibition of intrinsic apoptosis in U2-OS cells expressing VACV anti-apoptotic proteins

**Fig. 3.**
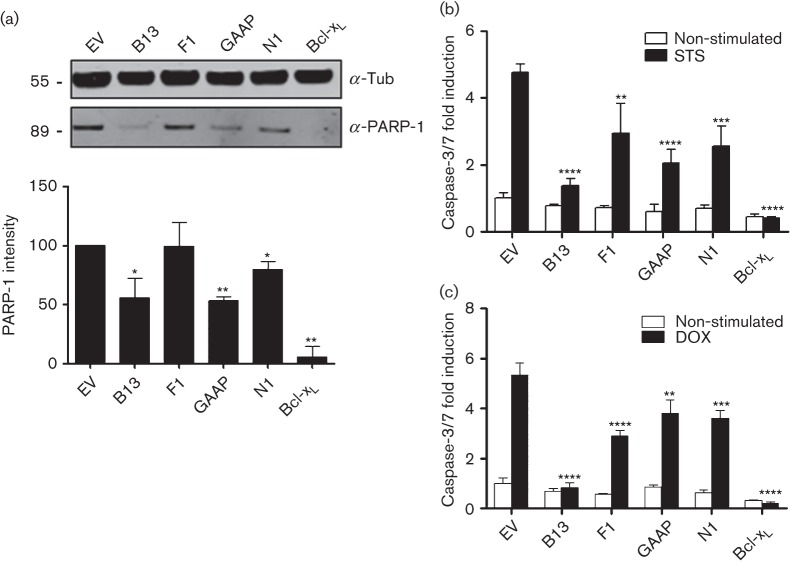
Induction of intrinsic apoptosis in the transduced U2-OS cells. (a) Cells were incubated with STS (0.5 µM) for 8 h. Cell lysates were resolved by SDS-PAGE and analysed by immunoblotting against cleaved PARP-1. A representative blot of three repeats is shown. Quantitative data were obtained by integrating the intensity of the bands from the three repeats using a LI-COR system. Tub, α-tubulin. Molecular size markers are indicated on the left (kDa). (b, c) Cells were treated with STS for 8 h (b) or DOX (3 µM) for 30 h (c) and apoptosis was assessed quantitatively using Caspase-Glo. Data are shown as the mean±sd fold induction relative to EV and are representative of at least three experiments each performed in triplicate. Statistical differences between EV and anti-apoptotic protein-transduced cells were determined using an unpaired Student’s *t*-test (*P<0.05, **P<0.01, ***P<0.001, ****P<0.0001).

Next, the resistance of these cell lines to intrinsic apoptosis induced by staurosporine (STS) was investigated. This showed that B13 caused substantial reduction in cleavage of PARP-1 compared with EV-transduced cells, whereas cells expressing GAAP and N1 showed a milder effect (Fig. 3a). Quantitative analysis demonstrated significant reduction by all these VACV proteins except F1 (Fig. 3a). Using the Caspase-Glo 3/7 assay, STS treatment induced around fivefold increase in caspase-3/7 activity in EV-transduced cells and this was reduced by each VACV protein, particularly B13 (Fig. 3b). Likewise, around fivefold increase in active caspase-3 was observed in EV-transduced cell lines upon treatment with doxorubicin (DOX), a DNA-damaging agent causing mitochondrial apoptosis, and this was reduced to different degrees by B13, F1, N1 or GAAP (Fig. 3c). Taken together, each VACV anti-apoptotic protein was sufficient to reduce or inhibit drug-induced intrinsic apoptosis in U2-OS cells.

### Induction of apoptosis using vv811

**Fig. 4.**
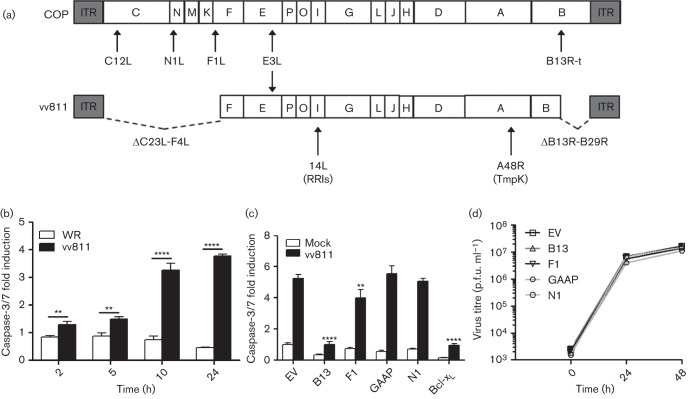
vv811 infection of U2-OS cell lines expressing VACV anti-apoptotic proteins. (a) Schematic of the genome structure of VACV strains COP and vv811, with the position of the anti-apoptotic proteins indicated. ITR, inverted terminal repeat; B13R-t, truncated B13R gene; RRls, ribonucleotide reductase large subunit; TmpK, thymidylate kinase. Note the C12L gene of VACV strain COP is equivalent to the B22R gene of VACV strain WR. (b) U2-OS parental cells were infected at 2.5 p.f.u. per cell with WR or vv811 and caspase-3/7 activity was assessed when indicated using Caspase-Glo. Data were normalized to mock-infected cells to obtain a fold induction. (c) U2-OS cell lines were infected with vv811 at 2.5 p.f.u. per cell for 24 h and apoptosis was assessed quantitatively using Caspase-Glo. Data are shown as the mean±sd and are representative of at least three experiments performed in triplicate. Statistical differences were determined using an unpaired Student’s *t*-test (*P<0.05, **P<0.01, ****P<0.0001). (d) Cells were infected with vv811 at 0.1 p.f.u. per cell, harvested at the indicated time and the intracellular virus titre was determined by plaque assay on BSC-1 cells.

VACV strain vv811 lacks 55 genes, including all known VACV anti-apoptotic proteins except E3 (Fig. 4a), and so we hypothesized that vv811 infection might trigger apoptosis without additional stimuli. To examine this, the level of caspase-3/7 activation in U2-OS cells infected with vv811 or WR was measured. VACV strain vv811 infection induced caspase-3/7 activation from 2 h post-infection (p.i.) and this increased over 24 h (Fig. 4b), whereas apoptosis was not induced by VACV strains WR (Fig. 4b) or COP (data not shown). The use of vv811 as an inducer of apoptosis provided a more physiological context to assess apoptosis without the need for exogenous drug treatments.

Next, the ability of the individual VACV anti-apoptotic proteins to inhibit apoptosis induced by vv811 and to enhance the poor growth of vv811 was examined. First, U2-OS cell lines expressing VACV proteins were infected for 24 h and the levels of apoptosis assessed by measuring caspase-3/7 activity using Caspase-Glo (Fig. 4c). F1 and B13 reduced vv811-induced apoptosis, whereas GAAP and N1 did not. Secondly, we produced multi-step growth curves of vv811 in the different U2-OS cell lines. Expression *in trans* of VACV anti-apoptotic proteins did not enhance viral growth (Fig. 4d). Taken together, these data indicated growth of vv811 was not enhanced in U2-OS cell lines expressing VACV proteins irrespective of their ability to prevent vv811-induced apoptosis.

### Generation of recombinant vv811 expressing VACV anti-apoptotic proteins

**Fig. 5.**
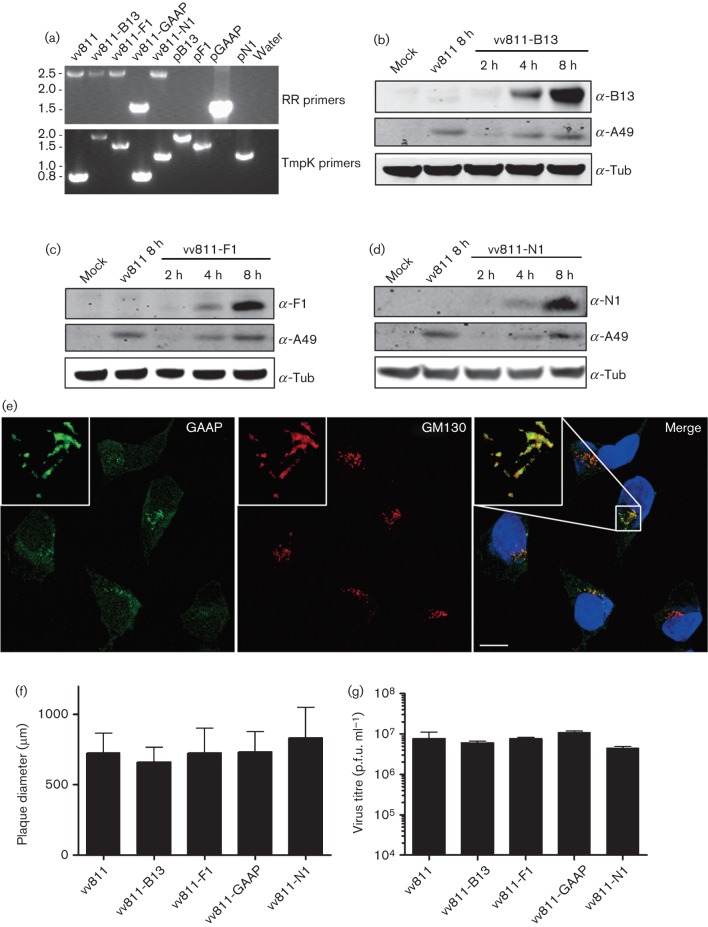
Construction of recombinant vv811 viruses. (a) The genotypes of the resolved viruses were analysed by PCR from proteinase K-treated, infected BSC-1 cell lysates using primers annealing to the flanking regions of the RR or TmpK genes. The resulting PCR products were compared with those obtained from plasmid templates (pB13, pF1, pGAAP and pN1). Molecular size markers are indicated on the left (kbp). (b–d) BSC-1 cells were infected with 5 p.f.u. per cell of the indicated recombinant or parental vv811 viruses and cell lysates were analysed by immunobloting using specific polyclonal antiserum for B13 (b), F1 (c) and N1 (d), as well as A49 and α-tubulin (Tub). (e) U2-OS cells were infected for 8 h with 2.5 p.f.u. per cell of vv811-GAAP and stained using a polyclonal antiserum for GAAP and antibody to GM130. DNA was stained with DAPI. Bar, 20 µm. A magnified image of the small square from the merged image is shown on the top left of each panel. (f) U2-OS cells were infected with the indicated viruses for 96 h. Cells were stained with crystal violet and the radius of >15 plaques per virus was measured. Data are expressed as the mean±sd plaque radius (μm). (g) U2-OS cells were infected with the indicated viruses at 2.5 p.f.u. per cell. Cells were harvested into the medium at 24 h and the virus titre was determined by plaque assay on U2-OS cells. Data are shown as the mean±sd.

To address whether expression of VACV anti-apoptotic proteins *in cis* could provide stronger effects, we generated recombinant vv811 expressing B13, F1, N1 or GAAP. To allow us to compare the efficiency of each protein independently of its expression level, each gene was cloned downstream of the VACV early/late promoter P7.5 (Mackett *et al.*, 1984) and inserted by transient dominant selection into vv811 within non-essential genes encoding either the ribonucleotide reductase large subunit (I4L gene) (Tengelsen *et al.*, 1988) or the thymidylate kinase (A48R gene) (Smith *et al.*, 1989a) as described in Methods. The genomes of the derivative vv811 viruses were shown by PCR using primers annealed to the flanking regions to contain the inserted genes as expected (Fig. 5a). Expression of the inserted genes was confirmed by immunoblotting with specific antibody for B13 (Fig. 5b), F1 (Fig. 5c) and N1 (Fig. 5d), and by immunofluorescence for GAAP (Fig. 5e). For B13, F1 and N1, expression was detected as early as 2–4 h p.i., in agreement with the expression from the 7.5K promoter (Mackett *et al.*, 1984), and was not detected in the vv811 parental strain. Equal infection of cells was confirmed by blotting for the VACV early protein A49 (Mansur *et al.*, 2013). The localization of GAAP in the Golgi was shown by co-staining with GM130, a marker of the Golgi complex (Fig. 5e). The recombinant viruses were characterized further by measuring plaque sizes in U2-OS cells after 96 h of infection (Fig. 5f) and by assessing viral replication in U2-OS cells infected for 24 h at 2.5 p.f.u. per cell (Fig. 5g). No statistically significant differences amongst the four recombinant viruses, and between these and the parental vv811, were observed in either of the assays. Taken together, these data indicated that the expression of these proteins *in cis* did not enhance viral replication or spread in the conditions tested.

### Induction of apoptosis by the recombinant vv811 viruses

The ability of these anti-apoptotic proteins to inhibit apoptosis during VACV infection was investigated using either VACV strains that lack each gene individually or vv811 viruses engineered to express each gene individually. Viruses lacking the B13R (Kettle *et al.*, 1995) and N1L (Bartlett *et al.*, 2002) genes from the WR strain had been constructed previously, whereas a virus lacking the GAAP gene was engineered in the Evans strain, one of the few VACV strains to express GAAP (Gubser *et al.*, 2007), and a F1L gene deletion virus was produced in the same WR strain background as we had used previously (see Methods). Deletion of the F1L gene from WR was confirmed by PCR using primers that annealed to the flanking regions of the F1L gene and by immunoblotting with anti-F1 antiserum (Fig. S2).

**Fig. 6.**
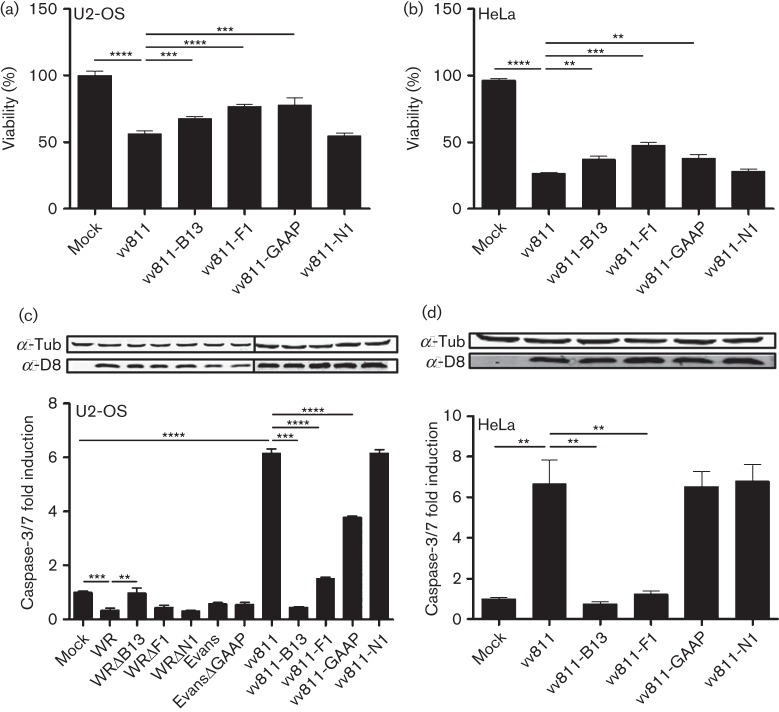
Induction of apoptosis by the recombinant vv811 viruses. (a, b) Cells were seeded in 96-well plates and infected at 2.5 p.f.u. per cell with recombinant vv811 viruses for 24 h. Cell viability was measured in U2-OS (a) and HeLa (b) cells using the CellTiter-Blue cell viability assay according to the manufacturer’s recommendations. (c, d) U2-OS (c) and HeLa (d) cells were infected with recombinant vv811 viruses and recombinant VACV generated in either strain WR (ΔB13, ΔF1 and ΔN1) or strain Evans (ΔGAAP) as in (a, b) and apoptosis was assessed quantitatively using Caspase-Glo. Cell lysates were analysed by SDS-PAGE and immunoblotting with antibodies against the indicated proteins. Data are shown as the mean±sd and are representative of at least three experiments performed in triplicate. Statistical differences were determined using an unpaired Student’s *t*-test (*P<0.05, **P<0.01, ***P<0.001, ****P<0.0001).

To determine the role of VACV anti-apoptotic proteins in both WT VACV strains and vv811, U2-OS cells were infected at 2.5 p.f.u. per cell and the cell viability was measured using the Cell Titre-Blue assay at 24 h p.i.. Infection with vv811 reduced viability by 50 %, and this was ameliorated by infection with vv811-B13, vv811-F1 and vv811-GAAP, but not vv811-N1 (Fig. 6a). Similar results were obtained in HeLa cells, where viability after vv811 infection reached only 25 % and could only be increased by B13, F1 or GAAP expression (Fig. 6b).

Next, caspase activity was measured. U2-OS cells were infected for 24 h with parental WR and its individual deletion derivatives WRΔB13, WRΔF1 and WRΔN1, with parental Evans and its derivative EvansΔGAAP, and with the recombinant vv811 viruses (Fig. 6c). The level of infection was confirmed by immunoblotting for VACV protein D8. As expected, neither VACV strain WR nor Evans induced apoptosis in this system. With the exception of WRΔB13, which induced significantly more caspase-3/7 activation than WR, deletion of each individual protein in each parental VACV strain did not change the level of caspase activity, presumably due to the presence of the other anti-apoptotic proteins. Conversely, after vv811 infection the anti-apoptotic effects of B13, F1 and GAAP were readily detectable, with each virus inducing significantly less apoptosis than parental vv811. As observed in assays where the proteins were expressed *in trans*, B13 was the most potent inhibitor. vv811-N1 did not, however, show any ability to block viral induction of caspase activation during infection. Furthermore, caspase activation after vv811 infection was also measured in HeLa cells (Fig. 6d), a cell line commonly used for apoptosis studies. Results were similar to those in U2-OS cells with the exception of vv811-GAAP, which did not show any protection in this cell type. Collectively, these data showed that inserting anti-apoptotic proteins individually in vv811 provided a powerful tool to assess their potency and relative contribution to preventing apoptosis in the context of viral infection, and that this system could be used in multiple cell types.

## Discussion

The ability of cells to undergo apoptosis is an important host antiviral mechanism that limits the replication and spread of many viruses and, consequently, viruses have evolved countermeasures. VACV encodes several anti-apoptotic proteins (see Introduction), but their anti-apoptotic activity has not been compared in the same experimental system and not in a context where apoptosis is induced by viral infection alone. In this study, we show that infection by VACV strain vv811, which unlike WT VACV strains lacks B13, B22, F1, N1 and GAAP proteins, induces apoptosis in cells without additional stimuli and so can be used to study the inhibitory activity of individual anti-apoptotic proteins in the context of viral infection. Of the four anti-apoptotic proteins studied (B13, F1, N1 and GAAP), B13 was found to be the most potent inhibitor.

When expressed in isolation, all four proteins were able to block apoptosis mediated by STS or DOX to some degree, whereas only B13 blocked apoptosis mediated by TNF-α (Figs 2 and 3). This is consistent with F1 and N1 targeting cellular Bcl-2 proteins involved in mitochondrial apoptotic pathways (Wasilenko *et al.*, 2003, 2005; Postigo *et al.*, 2006; Cooray *et al.*, 2007; Kvansakul *et al.*, 2008; Maluquer de Motes *et al.*, 2011), B13 being a pan-inhibitor of caspases (Ray *et al.*, 1992; Dobbelstein & Shenk, 1996; Kettle *et al.*, 1997; Garcia-Calvo *et al.*, 1998), and GAAP inhibiting intrinsic apoptosis (Gubser *et al.*, 2007). Although GAAP was reported to have some activity against extrinsic apoptosis (Gubser *et al.*, 2007), such activity was not found under the conditions tested here and this discrepancy might, in part, be explained by the efficiency of the method used to deliver GAAP into cells, which differed in these studies.

As vv811 infection is sufficient to induce apoptosis, the ability of the VACV proteins to block this was tested by infecting cell lines expressing these proteins and by constructing recombinant vv811 viruses expressing these proteins. After vv811 infection of the transduced cell lines, only B13 and F1 showed an anti-apoptotic effect (Fig. 4), indicating that B13 and F1 are more potent inhibitors than GAAP and N1 in this experimental system, and/or that vv811 infection triggered apoptotic responses in a manner that was not mimicked by the drugs employed in Fig. 3. Use of the recombinant vv811 viruses demonstrated that B13 and F1 had the greatest anti-apoptotic activity in both U2-OS and HeLa cells, highlighting their more potent inhibitory effects compared with GAAP and N1 (Fig. 6). Interestingly, infection of U2-OS cells with vv811-GAAP increased cell viability similar to vv811-B13 or vv811-F1, despite being unable to reduce caspase-3/7 activation similar to those viruses. Notably, in HeLa cells, viability data were recapitulated despite no reduction of caspase-3/7 activity. In fact, vv811-GAAP conferred a viability phenotype similar to vv811-F1 in U2-OS and similar to vv811-B13 in HeLa cells despite intermediate or null inhibition of caspase activity. This was confirmed by a very weak cytopathogenic effect after infection compared with other vv811 viruses (data not shown). Taken together, these data suggest that GAAP may contribute to cell viability by multiple mechanisms in addition to inhibition of caspase activity, and this may be more prominent in specific cell types. Indeed, the very closely related hGAAP has also been linked to cell adhesion and migration (Saraiva *et al.*, 2013a). In contrast, B13 proved to be the most potent anti-apoptotic protein under all the conditions tested. However, despite this drastic inhibition of apoptosis, vv811-B13 infection only increased cell viability mildly. Taken together, this indicates that caspase-3/7 inhibition is not sufficient to determine cell viability during infection and other factors are required.

Although the anti-apoptotic effect of N1 was observed in isolation upon drug treatment (Fig. 3), this was not mirrored in the context of viral infection (Fig. 6). N1 is a Bcl-2-like protein that in addition to modulating apoptosis, inhibits activation of the pro-inflammatory transcription factor NFκB (DiPerna *et al.*, 2004; Cooray *et al.*, 2007; Chen *et al.*, 2008; Graham *et al.*, 2008; Maluquer de Motes *et al.*, 2011). Recently, a structurally informed mutagenesis study demonstrated that it is the anti-NFκB activity of N1 and not its anti-apoptotic ability that contributes to virulence *in vivo* (Maluquer de Motes *et al.*, 2011). Therefore, although N1 has a relatively weak anti-apoptotic activity, its main function following infection is the inhibition of inflammatory signalling. Interestingly, a similar situation exists for F1 that contributes to virulence by targeting NLRP-1 and thereby blocking inflammasome activation (Gerlic *et al.*, 2013). Although F1 has greater anti-apoptotic activity than N1, virulence in both cases is driven by inhibition of innate immune signalling rather than apoptosis.

VACV is a large virus that alters the cellular environment by multiple mechanisms. Here, we demonstrate that unlike its parental full-length strain COP, infection with vv811 causes caspase-3/7 activation in cells. Thus, VACV infection induces apoptosis and this is prevented by anti-apoptotic proteins. Exactly how VACV infection is sensed to trigger apoptosis requires clarification. VACV strain COP lacks GAAP (Goebel *et al.*, 1990; Gubser & Smith, 2002) and has a non-functional B13R gene (Kettle *et al.*, 1995), indicating that these genes are non-essential for blocking apoptosis during COP infection. In contrast, proteins F1 and N1 are highly conserved amongst VACV strains and other orthopoxviruses, and each interferes with mitochondrial apoptosis, suggesting that this apoptotic route may be prominent during viral infection. Data presented here show, however, that only B13 reduced caspase-3/7 activation during vv811 infection to levels observed with VACV strains WR or COP. Given that the anti-apoptotic activities of N1 and F1 do not contribute to virulence, it is likely that caspase activation occurs *in vivo* primarily via non-mitochondrial pathways including Fas ligand or TNF signalling, and this is blocked by B13. Although a few VACV strains express soluble TNF receptors (Alcamí *et al.*, 1999), intracellular inhibitors of such extrinsic apoptosis other than B13 have not been described in VACV, but are present in other large DNA viruses, such as human cytomegalovirus (Skaletskaya *et al.*, 2001).

In summary, this study shows that vv811 infection induces apoptosis, and so is a useful tool to identify the cellular factors that trigger recognition and apoptosis upon VACV infection. Such information will be useful in understanding VACV–host interactions, but also for developing VACV as a vaccine vector and oncolytic agent.

## Methods

### 

#### Plasmids, cells and viruses.

The F1L and B13R genes were amplified by PCR from VACV strain WR, and cloned into pcDNA4/TO fused with an N-terminal tandem affinity purification (TAP) tag consisting of a streptavidin-binding sequence and FLAG epitope (Gloeckner *et al.*, 2007). GAAP was amplified by PCR from VACV strain Evans and cloned as the C-terminal TAP fusion. N1-TAP was described previously (Maluquer de Motes *et al.*, 2011). All plasmids were verified by DNA sequencing. Finally, tagged alleles, as well as Bcl-x_L_, were subcloned into the first cistron of a lentivirus vector also expressing GFP (Saraiva *et al.*, 2013b). BSC-1 and U2-OS cells were grown in Dulbecco’s modified Eagle’s medium (DMEM) supplemented with 10 % FBS (Biosera), 100 U penicillin ml^−1^ (Invitrogen) and 100 mg streptomycin ml^−1^ (Invitrogen). HeLa cells were grown in minimum essential medium (Gibco) supplemented as above plus non-essential amino acids (Sigma). U2-OS cells expressing proteins B13, F1, GAAP, N1 or Bcl-x_L_, or EV control, were obtained after transduction with lentiviruses expressing GFP and the protein of interest, and sorting in a MoFlo MLS high-speed cell sorter (Beckman Coulter) to obtain >95 % GFP-positive cells. Lentivirus particles were generated after transient transfection of HEK 293T cells with the bicistronic genomic vector together with entry and packaging vectors using the calcium chloride method. Parental vv811 was provided by Michelle Barry (University of Alberta, Canada). VACV strain WR lacking either B13R or N1L and VACV strain Evans lacking GAAP have been described (Kettle *et al.*, 1995; Bartlett *et al.*, 2002; Gubser *et al.*, 2007).

#### Construction of mutant viruses.

For the construction of vv811-B13, vv811-F1 and vv811-N1, plasmids containing the B13R, F1L or N1L gene flanked by ~250 bp regions of the A48R gene encoding thymidylate kinase (Smith *et al.*, 1989a; Hughes *et al.*, 1991) and containing the *Escherichia coli* guanylphosphoribosyltransferase gene (*Ecogpt*) fused in-frame with the EGFP gene were used (Ember *et al.*, 2012). For the construction of vv811-GAAP, a similar plasmid was used containing the GAAP gene inserted between the flanking regions of the I4L gene encoding the ribonucleotide reductase large subunit (Tengelsen *et al.*, 1988). VACV strain WR lacking the F1L gene (WRΔF1) was constructed using a similar plasmid containing the 250 bp flanking region of the F1L gene. The mutant viruses were constructed using the transient dominant selection method (Falkner & Moss, 1990) by selection of EGFP-positive plaques in the presence of mycophenolic acid, hypoxanthine and xanthine, as described previously (Unterholzner *et al.*, 2011). The genotype of the resolved viruses was analysed by PCR following proteinase K treatment of infected BSC-1 cells using primers that annealed within the flanking regions of each gene.

#### Immunofluorescence.

Transduced U2-OS cells were seeded into six-well plates containing sterile glass coverslips. After two washes with ice-cold PBS, cells were fixed in 4 % (v/v) paraformaldehyde, quenched in 150 mM ammonium chloride, permeabilized with 0.1 % (v/v) Triton X-100 in PBS and blocked for 30 min in 5 % (w/v) FBS in PBS. The cells were stained with rabbit anti-FLAG antibody (Sigma) for 1 h, followed by incubation with goat anti-rabbit Alexa Fluor 546 secondary antibody (Invitrogen). Coverslips were mounted in MOWIOL 4-88 (Calbiochem) containing DAPI. Images were taken on a Zeiss LSM780 confocal microscope using Zen2011 acquisition software. Following vv811-GAAP infection, U2-OS cells were stained using rabbit anti-GAAP polyclonal antiserum (Gubser *et al.*, 2007) and mouse anti-GM130 antibody (BD Biosciences) to check GAAP expression and localization.

#### Plaque assays and growth curves.

U2-OS cells were infected with vv811 and derivative viruses at 50 p.f.u. per well of a six-well plate for 5 days. The cells were washed once with PBS and stained for 1 h with crystal violet [5 % (v/v) crystal violet solution (Sigma), 25 % (v/v) ethanol]. The sizes of 20 plaques per well were measured using Axiovision acquisition software and a Zeiss AxioVert.A1 inverted microscope as described previously (Doceul *et al.*, 2010; Ferguson *et al.*, 2013). To measure virus growth, U2-OS cell lines were infected with vv811 at 0.1 p.f.u. per cell, and samples harvested at 0, 24 and 48 h. U2-OS cells were infected with recombinant viruses at 2.5 p.f.u. per cell for 24 h. Virus titres were determined as described by Chen *et al.* (2006), but using U2-OS cells.

#### Apoptosis and viability assay.

Apoptosis was induced with STS (0.5 µM, 8 h), DOX (3 µM, 30 h) or human TNF-α (10 µg ml^−1^; Peprotech) together with CHX (30 µg ml^−1^) for 16 h. Virus-induced apoptosis was analysed after infection with 2.5 p.f.u. per cell for 24 h. Cleaved PARP-1 was analysed by immunoblotting from six-well plates. Caspase-3 activity was measured in 96-well plates using Caspase-Glo 3/7 (Promega). Cell viability was measured in 96-well plates using CellTiter-Blue (Promega) according to the manufacturer’s recommendations.

#### SDS-PAGE and immunobloting.

Cells were lysed with ice-cold lysis buffer (50 mM Tris/HCl, pH 7.5, 100 mM NaCl, 1 % CHAPS (w/v) and protease inhibitor (Roche)]. The samples were resolved by SDS-PAGE and transferred onto nitrocellulose membranes. Primary antibodies were mouse anti-α-tubulin (Upstate Biotech), mouse anti-FLAG (Sigma) and mouse anti-PARP-1 (Cell Signalling). Antibody against VACV proteins D8 (Parkinson & Smith, 1991), A49 (Mansur *et al.*, 2013), B13 (Kettle *et al.*, 1995), F1 (Postigo *et al.*, 2006) and N1 (Bartlett *et al.*, 2002) were as described. Primary antibodies were detected using IRDye-conjugated secondary antibodies and an Odyssey Infrared Imager (LI-COR Biosciences). Quantitative analysis was obtained by integration of the band intensity using Odyssey software.

#### Statistical analysis.

Data were analysed using an unpaired Student’s *t*-test, with Welch’s correction where appropriate.

## References

[r1] Alcamí A. Khanna A. Paul N. L. Smith G. L. **(**1999**).** Vaccinia virus strains Lister, USSR and Evans express soluble and cell-surface tumour necrosis factor receptors. J Gen Virol 80, 949–959. 10211965 10.1099/0022-1317-80-4-949

[r2] Ali A. N. Turner P. C. Brooks M. A. Moyer R. W. **(**1994**).** The SPI-1 gene of rabbitpox virus determines host range and is required for hemorrhagic pock formation. Virology 202, 305–314. 10.1006/viro.1994.1347 8009842

[r3] Aoyagi M. Zhai D. Jin C. Aleshin A. E. Stec B. Reed J. C. Liddington R. C. **(**2007**).** Vaccinia virus N1L protein resembles a B cell lymphoma-2 (Bcl-2) family protein. Protein Sci 16, 118–124. 10.1110/ps.062454707 17123957 PMC2222835

[r4] Banadyga L. Veugelers K. Campbell S. Barry M. **(**2009**).** The fowlpox virus BCL-2 homologue, FPV039, interacts with activated Bax and a discrete subset of BH3-only proteins to inhibit apoptosis. J Virol 83, 7085–7098. 10.1128/JVI.00437-09 19439472 PMC2704773

[r5] Bartlett N. Symons J. A. Tscharke D. C. Smith G. L. **(**2002**).** The vaccinia virus N1L protein is an intracellular homodimer that promotes virulence. J Gen Virol 83, 1965–1976. 12124460 10.1099/0022-1317-83-8-1965

[r6] Brooks M. A. Ali A. N. Turner P. C. Moyer R. W. **(**1995**).** A rabbitpox virus serpin gene controls host range by inhibiting apoptosis in restrictive cells. J Virol 69, 7688–7698. 7494278 10.1128/jvi.69.12.7688-7698.1995 PMC189710

[r7] Carrara G. Saraiva N. Gubser C. Johnson B. F. Smith G. L. **(**2012**).** Six-transmembrane topology for Golgi anti-apoptotic protein (GAAP) and Bax inhibitor 1 (BI-1) provides model for the transmembrane Bax inhibitor-containing motif (TMBIM) family. J Biol Chem 287, 15896–15905. 10.1074/jbc.M111.336149 22418439 PMC3346125

[r8] Chen R. A. Jacobs N. Smith G. L. **(**2006**).** Vaccinia virus strain Western Reserve protein B14 is an intracellular virulence factor. J Gen Virol 87, 1451–1458. 10.1099/vir.0.81736-0 16690909

[r9] Chen R. A. Ryzhakov G. Cooray S. Randow F. Smith G. L. **(**2008**).** Inhibition of IkappaB kinase by vaccinia virus virulence factor B14. PLoS Pathog 4, e22. 10.1371/journal.ppat.0040022 18266467 PMC2233672

[r10] Cho Y. S. Challa S. Moquin D. Genga R. Ray T. D. Guildford M. Chan F. K. **(**2009**).** Phosphorylation-driven assembly of the RIP1–RIP3 complex regulates programmed necrosis and virus-induced inflammation. Cell 137, 1112–1123. 10.1016/j.cell.2009.05.037 19524513 PMC2727676

[r11] Cooray S. Bahar M. W. Abrescia N. G. McVey C. E. Bartlett N. W. Chen R. A. Stuart D. I. Grimes J. M. Smith G. L. **(**2007**).** Functional and structural studies of the vaccinia virus virulence factor N1 reveal a Bcl-2-like anti-apoptotic protein. J Gen Virol 88, 1656–1666. 10.1099/vir.0.82772-0 17485524 PMC2885619

[r12] Dai P. Wang W. Cao H. Avogadri F. Dai L. Drexler I. Joyce J. A. Li X. D. Chen Z. **& other authors (**2014**).** Modified vaccinia virus Ankara triggers type I IFN production in murine conventional dendritic cells via a cGAS/STING-mediated cytosolic DNA-sensing pathway. PLoS Pathog 10, e1003989. 10.1371/journal.ppat.1003989 24743339 PMC3990710

[r13] de Mattia F. Gubser C. van Dommelen M. M. Visch H. J. Distelmaier F. Postigo A. Luyten T. Parys J. B. de Smedt H. **& other authors (**2009**).** Human Golgi antiapoptotic protein modulates intracellular calcium fluxes. Mol Biol Cell 20, 3638–3645. 10.1091/mbc.E09-05-0385 19553469 PMC2777924

[r14] DiPerna G. Stack J. Bowie A. G. Boyd A. Kotwal G. Zhang Z. Arvikar S. Latz E. Fitzgerald K. A. Marshall W. L. **(**2004**).** Poxvirus protein N1L targets the I-kappaB kinase complex, inhibits signaling to NF-kappaB by the tumor necrosis factor superfamily of receptors, and inhibits NF-kappaB and IRF3 signaling by toll-like receptors. J Biol Chem 279, 36570–36578. 10.1074/jbc.M400567200 15215253

[r15] Dobbelstein M. Shenk T. **(**1996**).** Protection against apoptosis by the vaccinia virus SPI-2 (B13R) gene product. J Virol 70, 6479–6485. 8709286 10.1128/jvi.70.9.6479-6485.1996 PMC190684

[r16] Doceul V. Hollinshead M. van der Linden L. Smith G. L. **(**2010**).** Repulsion of superinfecting virions: a mechanism for rapid virus spread. Science 327, 873–876. 10.1126/science.1183173 20093437 PMC4202693

[r17] Ember S. W. Ren H. Ferguson B. J. Smith G. L. **(**2012**).** Vaccinia virus protein C4 inhibits NF-κB activation and promotes virus virulence. J Gen Virol 93, 2098–2108. 10.1099/vir.0.045070-0 22791606 PMC3541790

[r18] Enari M. Hug H. Nagata S. **(**1995**).** Involvement of an ICE-like protease in Fas-mediated apoptosis. Nature 375, 78–81. 10.1038/375078a0 7536900

[r19] Falkner F. G. Moss B. **(**1990**).** Transient dominant selection of recombinant vaccinia viruses. J Virol 64, 3108–3111. 2159565 10.1128/jvi.64.6.3108-3111.1990 PMC249504

[r20] Ferguson B. J. Benfield C. T. Ren H. Lee V. H. Frazer G. L. Strnadova P. Sumner R. P. Smith G. L. **(**2013**).** Vaccinia virus protein N2 is a nuclear IRF3 inhibitor that promotes virulence. J Gen Virol 94, 2070–2081. 10.1099/vir.0.054114-0 23761407 PMC3749055

[r21] García M. A. Guerra S. Gil J. Jimenez V. Esteban M. **(**2002**).** Anti-apoptotic and oncogenic properties of the dsRNA-binding protein of vaccinia virus, E3L. Oncogene 21, 8379–8387. 10.1038/sj.onc.1206036 12466958

[r22] Garcia-Calvo M. Peterson E. P. Leiting B. Ruel R. Nicholson D. W. Thornberry N. A. **(**1998**).** Inhibition of human caspases by peptide-based and macromolecular inhibitors. J Biol Chem 273, 32608–32613. 10.1074/jbc.273.49.32608 9829999

[r23] Gerlic M. Faustin B. Postigo A. Yu E. C. Proell M. Gombosuren N. Krajewska M. Flynn R. Croft M. **& other authors (**2013**).** Vaccinia virus F1L protein promotes virulence by inhibiting inflammasome activation. Proc Natl Acad Sci U S A 110, 7808–7813. 10.1073/pnas.1215995110 23603272 PMC3651467

[r24] Gloeckner C. J. Boldt K. Schumacher A. Roepman R. Ueffing M. **(**2007**).** A novel tandem affinity purification strategy for the efficient isolation and characterisation of native protein complexes. Proteomics 7, 4228–4234. 10.1002/pmic.200700038 17979178

[r25] Goebel S. J. Johnson G. P. Perkus M. E. Davis S. W. Winslow J. P. Paoletti E. **(**1990**).** The complete DNA sequence of vaccinia virus. Virology 179, 247–266. 10.1016/0042-6822(90)90294-2 2219722

[r26] Graham S. C. Bahar M. W. Cooray S. Chen R. A. Whalen D. M. Abrescia N. G. Alderton D. Owens R. J. Stuart D. I. **& other authors (**2008**).** Vaccinia virus proteins A52 and B14 Share a Bcl-2-like fold but have evolved to inhibit NF-kappaB rather than apoptosis. PLoS Pathog 4, e1000128. 10.1371/journal.ppat.1000128 18704168 PMC2494871

[r27] Griffith T. S. Ferguson T. A. **(**2011**).** Cell death in the maintenance and abrogation of tolerance: the five Ws of dying cells. Immunity 35, 456–466. 10.1016/j.immuni.2011.08.011 22035838 PMC3205359

[r28] Gubser C. Smith G. L. **(**2002**).** The sequence of camelpox virus shows it is most closely related to variola virus, the cause of smallpox. J Gen Virol 83, 855–872. 11907336 10.1099/0022-1317-83-4-855

[r29] Gubser C. Hué S. Kellam P. Smith G. L. **(**2004**).** Poxvirus genomes: a phylogenetic analysis. J Gen Virol 85, 105–117. 10.1099/vir.0.19565-0 14718625

[r30] Gubser C. Bergamaschi D. Hollinshead M. Lu X. van Kuppeveld F. J. Smith G. L. **(**2007**).** A new inhibitor of apoptosis from vaccinia virus and eukaryotes. PLoS Pathog 3, e17. 10.1371/journal.ppat.0030017 17319741 PMC1803007

[r31] Han J. Zhong C. Q. Zhang D. W. **(**2011**).** Programmed necrosis: backup to and competitor with apoptosis in the immune system. Nat Immunol 12, 1143–1149. 10.1038/ni.2159 22089220

[r32] Heinkelein M. Pilz S. Jassoy C. **(**1996**).** Inhibition of CD95 (Fas/Apo1)-mediated apoptosis by vaccinia virus WR. Clin Exp Immunol 103, 8–14. 10.1046/j.1365-2249.1996.927619.x 8565292 PMC2200317

[r33] Holler N. Zaru R. Micheau O. Thome M. Attinger A. Valitutti S. Bodmer J. L. Schneider P. Seed B. Tschopp J. **(**2000**).** Fas triggers an alternative, caspase-8-independent cell death pathway using the kinase RIP as effector molecule. Nat Immunol 1, 489–495. 10.1038/82732 11101870

[r34] Hughes S. J. Johnston L. H. de Carlos A. Smith G. L. **(**1991**).** Vaccinia virus encodes an active thymidylate kinase that complements a cdc8 mutant of *Saccharomyces cerevisiae*. J Biol Chem 266, 20103–20109. 1657913

[r35] Kettle S. Blake N. W. Law K. M. Smith G. L. **(**1995**).** Vaccinia virus serpins B13R (SPI-2) and B22R (SPI-1) encode M_r_ 38.5 and 40K, intracellular polypeptides that do not affect virus virulence in a murine intranasal model. Virology 206, 136–147. 10.1016/S0042-6822(95)80028-X 7831769

[r36] Kettle S. Alcamí A. Khanna A. Ehret R. Jassoy C. Smith G. L. **(**1997**).** Vaccinia virus serpin B13R (SPI-2) inhibits interleukin-1beta-converting enzyme and protects virus-infected cells from TNF- and Fas-mediated apoptosis, but does not prevent IL-1beta-induced fever. J Gen Virol 78, 677–685. 9049422 10.1099/0022-1317-78-3-677

[r37] Kibler K. V. Shors T. Perkins K. B. Zeman C. C. Banaszak M. P. Biesterfeldt J. Langland J. O. Jacobs B. L. **(**1997**).** Double-stranded RNA is a trigger for apoptosis in vaccinia virus-infected cells. J Virol 71, 1992–2003. 9032331 10.1128/jvi.71.3.1992-2003.1997 PMC191284

[r38] Kotwal G. J. Moss B. **(**1989**).** Vaccinia virus encodes two proteins that are structurally related to members of the plasma serine protease inhibitor superfamily. J Virol 63, 600–606. 2783466 10.1128/jvi.63.2.600-606.1989 PMC247729

[r39] Kvansakul M. Yang H. Fairlie W. D. Czabotar P. E. Fischer S. F. Perugini M. A. Huang D. C. Colman P. M. **(**2008**).** Vaccinia virus anti-apoptotic F1L is a novel Bcl-2-like domain-swapped dimer that binds a highly selective subset of BH3-containing death ligands. Cell Death Differ 15, 1564–1571. 10.1038/cdd.2008.83 18551131

[r40] Lee S. B. Esteban M. **(**1994**).** The interferon-induced double-stranded RNA-activated protein kinase induces apoptosis. Virology 199, 491–496. 10.1006/viro.1994.1151 7510087

[r41] Los M. Van de Craen M. Penning L. C. Schenk H. Westendorp M. Baeuerle P. A. Dröge W. Krammer P. H. Fiers W. Schulze-Osthoff K. **(**1995**).** Requirement of an ICE/CED-3 protease for Fas/APO-1-mediated apoptosis. Nature 375, 81–83. 10.1038/375081a0 7536901

[r42] Mackett M. Smith G. L. Moss B. **(**1984**).** General method for production and selection of infectious vaccinia virus recombinants expressing foreign genes. J Virol 49, 857–864. 6321770 10.1128/jvi.49.3.857-864.1984 PMC255547

[r43] Maluquer de Motes C. Cooray S. Ren H. Almeida G. M. F. McGourty K. Bahar M. W. Stuart D. I. Grimes J. M. Graham S. C. Smith G. L. **(**2011**).** Inhibition of apoptosis and NF-κB activation by vaccinia protein N1 occur via distinct binding surfaces and make different contributions to virulence. PLoS Pathog 7, e1002430. 10.1371/journal.ppat.1002430 22194685 PMC3240604

[r44] Mansur D. S. Maluquer de Motes C. Unterholzner L. Sumner R. P. Ferguson B. J. Ren H. Strnadova P. Bowie A. G. Smith G. L. **(**2013**).** Poxvirus targeting of E3 ligase β-TrCP by molecular mimicry: a mechanism to inhibit NF-κB activation and promote immune evasion and virulence. PLoS Pathog 9, e1003183. 10.1371/journal.ppat.1003183 23468625 PMC3585151

[r45] Miura M. Friedlander R. M. Yuan J. **(**1995**).** Tumor necrosis factor-induced apoptosis is mediated by a CrmA-sensitive cell death pathway. Proc Natl Acad Sci U S A 92, 8318–8322. 10.1073/pnas.92.18.8318 7667287 PMC41148

[r46] Perkus M. E. Goebel S. J. Davis S. W. Johnson G. P. Norton E. K. Paoletti E. **(**1991**).** Deletion of 55 open reading frames from the termini of vaccinia virus. Virology 180, 406–410. 10.1016/0042-6822(91)90047-F 1984660

[r47] Postigo A. Way M. **(**2012**).** The vaccinia virus-encoded Bcl-2 homologues do not act as direct Bax inhibitors. J Virol 86, 203–213. 10.1128/JVI.05817-11 22013032 PMC3255923

[r48] Postigo A. Cross J. R. Downward J. Way M. **(**2006**).** Interaction of F1L with the BH3 domain of Bak is responsible for inhibiting vaccinia-induced apoptosis. Cell Death Differ 13, 1651–1662. 10.1038/sj.cdd.4401853 16439990

[r49] Ray C. A. Black R. A. Kronheim S. R. Greenstreet T. A. Sleath P. R. Salvesen G. S. Pickup D. J. **(**1992**).** Viral inhibition of inflammation: cowpox virus encodes an inhibitor of the interleukin-1 beta converting enzyme. Cell 69, 597–604. 10.1016/0092-8674(92)90223-Y 1339309

[r50] Saraiva N. Prole D. L. Carrara G. Johnson B. F. Taylor C. W. Parsons M. Smith G. L. **(**2013a**).** hGAAP promotes cell adhesion and migration via the stimulation of store-operated Ca^2+^ entry and calpain 2. J Cell Biol 202, 699–713. 10.1083/jcb.201301016 23940116 PMC3747308

[r51] Saraiva N. Prole D. L. Carrara G. Maluquer de Motes C. Johnson B. F. Byrne B. Taylor C. W. Smith G. L. **(**2013b**).** Human and viral Golgi anti-apoptotic proteins (GAAPs) oligomerize via different mechanisms and monomeric GAAP inhibits apoptosis and modulates calcium. J Biol Chem 288, 13057–13067. 10.1074/jbc.M112.414367 23508950 PMC3642348

[r52] Shisler J. L. Moss B. **(**2001**).** Immunology 102 at poxvirus U: avoiding apoptosis. Semin Immunol 13, 67–72. 10.1006/smim.2000.0297 11289801

[r53] Skaletskaya A. Bartle L. M. Chittenden T. McCormick A. L. Mocarski E. S. Goldmacher V. S. **(**2001**).** A cytomegalovirus-encoded inhibitor of apoptosis that suppresses caspase-8 activation. Proc Natl Acad Sci U S A 98, 7829–7834. 10.1073/pnas.141108798 11427719 PMC35427

[r54] Smith G. L. de Carlos A. Chan Y. S. **(**1989a**).** Vaccinia virus encodes a thymidylate kinase gene: sequence and transcriptional mapping. Nucleic Acids Res 17, 7581–7590. 10.1093/nar/17.19.7581 2552411 PMC334868

[r55] Smith G. L. Howard S. T. Chan Y. S. **(**1989b**).** Vaccinia virus encodes a family of genes with homology to serine proteinase inhibitors. J Gen Virol 70, 2333–2343. 10.1099/0022-1317-70-9-2333 2778436

[r56] Smith G. L. Chan Y. S. Howard S. T. **(**1991**).** Nucleotide sequence of 42 kbp of vaccinia virus strain WR from near the right inverted terminal repeat. J Gen Virol 72, 1349–1376. 10.1099/0022-1317-72-6-1349 2045793

[r57] Smith G. L. Benfield C. T. Maluquer de Motes C. Mazzon M. Ember S. W. Ferguson B. J. Sumner R. P. **(**2013**).** Vaccinia virus immune evasion: mechanisms, virulence and immunogenicity. J Gen Virol 94, 2367–2392. 10.1099/vir.0.055921-0 23999164

[r58] Tait S. W. Green D. R. **(**2010**).** Mitochondria and cell death: outer membrane permeabilization and beyond. Nat Rev Mol Cell Biol 11, 621–632. 10.1038/nrm2952 20683470

[r59] Taylor J. M. Barry M. **(**2006**).** Near death experiences: poxvirus regulation of apoptotic death. Virology 344, 139–150. 10.1016/j.virol.2005.09.032 16364745

[r60] Tengelsen L. A. Slabaugh M. B. Bibler J. K. Hruby D. E. **(**1988**).** Nucleotide sequence and molecular genetic analysis of the large subunit of ribonucleotide reductase encoded by vaccinia virus. Virology 164, 121–131. 10.1016/0042-6822(88)90627-7 3284177

[r61] Tewari M. Dixit V. M. **(**1995**).** Fas- and tumor necrosis factor-induced apoptosis is inhibited by the poxvirus *crmA* gene product. J Biol Chem 270, 3255–3260. 10.1074/jbc.270.7.3255 7531702

[r62] Unterholzner L. Sumner R. P. Baran M. Ren H. Mansur D. S. Bourke N. M. Randow F. Smith G. L. Bowie A. G. **(**2011**).** Vaccinia virus protein C6 is a virulence factor that binds TBK-1 adaptor proteins and inhibits activation of IRF3 and IRF7. PLoS Pathog 7, e1002247. 10.1371/journal.ppat.1002247 21931555 PMC3169548

[r63] Wasilenko S. T. Meyers A. F. Vander Helm K. Barry M. **(**2001**).** Vaccinia virus infection disarms the mitochondrion-mediated pathway of the apoptotic cascade by modulating the permeability transition pore. J Virol 75, 11437–11448. 10.1128/JVI.75.23.11437-11448.2001 11689625 PMC114730

[r64] Wasilenko S. T. Stewart T. L. Meyers A. F. Barry M. **(**2003**).** Vaccinia virus encodes a previously uncharacterized mitochondrial-associated inhibitor of apoptosis. Proc Natl Acad Sci U S A 100, 14345–14350. 10.1073/pnas.2235583100 14610284 PMC283594

[r65] Wasilenko S. T. Banadyga L. Bond D. Barry M. **(**2005**).** The vaccinia virus F1L protein interacts with the proapoptotic protein Bak and inhibits Bak activation. J Virol 79, 14031–14043. 10.1128/JVI.79.22.14031-14043.2005 16254338 PMC1280199

